# Pulmonary Necrobiotic Nodules at Time of Diagnosis in a Patient with Ulcerative Colitis

**DOI:** 10.7759/cureus.7474

**Published:** 2020-03-30

**Authors:** Alec Kellish, Victoria Soal, Elizabeth Caskey, Gabrielle Hassinger, Nicole Terrigno

**Affiliations:** 1 Orthopaedics, Cooper Medical School of Rowan University, Camden, USA; 2 Psychiatry, Cooper Medical School of Rowan University, Camden, USA; 3 Internal Medicine, Cooper University Hospital, Camden, USA

**Keywords:** ulcerative colitis, inflammatory bowel disease, pulmonary nodules, extraintestinal manifestations, appendicitis

## Abstract

Inflammatory bowel disease (IBD) is an umbrella term encompassing intestinal conditions Crohn's disease and ulcerative colitis (UC), characterized by inflammation of intestinal wall, differing in location, depth, pathophysiology, and sequela. Extraintestinal manifestations (EIM) of IBD commonly affect the skin, joints, eyes, and in rare instances, the lungs. Pulmonary involvement affects the large and small airways, serosal surface, and vasculature leading to a range of pathology, including bronchiectasis, pleural effusions, and necrobiotic nodules. The presence of EIM is uncommon at the diagnosis, particularly in regard to pulmonary EIM, most commonly seen years later. We present the case of a 22-year-old African American female who was discovered to have pulmonary involvement of her UC at the time of her diagnosis complicating management of her appendicitis.

A 22-year-old female with a history of UC was transferred from an outside hospital for the management of her appendicitis after imaging revealed numerous pulmonary nodules. The presence of multiple cavitary pulmonary nodules delayed surgical intervention leading to a ruptured appendix. The patient had no cardiopulmonary complaints, and review of prior imaging studies showed these nodules to be present six weeks prior, the time of her diagnosis with UC. After antibiotic management failed, the patient required a laparotomy appendectomy with omentectomy due to resulting appendiceal abscess and phlegmon. The nodules were determined to be EIM of UC after sarcoidosis, infectious, and malignant etiologies were ruled out. For the pulmonary nodules, she is following at an outside hospital for the management of her UC as treatment of her underlying UC will result in a decrease or resolution of the EIM.

EIM of IBD may present in patients at any time, even before their initial diagnosis of an IBD. While pulmonary manifestations are rare EIM, the presence of pulmonary nodules at the time of initial diagnosis is exceedingly uncommon. Evaluation and management of these nodules, even if asymptomatic in nature, requires diligence and thorough documentation regarding their onset and etiology. In the event of a medical emergency, such as in the case of our patient with appendicitis, a lack of thorough documentation and evaluation of the nodules may result in unnecessary medical testing, invasive procedures, and delay in treatment of their current medical illness.

## Introduction

Inflammatory bowel disease (IBD) can result in extraintestinal manifestations (EIM) in virtually every organ system [1]. The EIM most commonly occur in the joints, skin, liver, and eyes, and effects approximately 17%-32% of individuals with IBD [2,3]. One of the rarest types of EIM is pulmonary involvement, particularly necrobiotic pulmonary nodules [4].

The association of pulmonary involvement with an IBD is believed to be due to the shared embryonic origins [5]. Both systems arise from the endoderm, an embryonic germ layer from which the digestive tub originates. As embryological development continues, a respiratory tube forms from the digestive tube, and then subsequently develops into the lungs [6]. Current theories suggest that sensitization occurring in epithelial cells lining the intestinal mucous propagates an inflammatory response that results in sensitization of pulmonary lymphoid tissue and inflammation within the lungs.

Pulmonary involvement from ulcerative colitis (UC) most commonly affects the larger airways, resulting in chronic bronchitis [7,8]. The subsequent inflammatory response ultimately results in bronchiectasis if left untreated [9]. Small airway involvement can occur but is less frequently than involvement of other parts of the lung, such as the serosal surface and lung parenchyma [10]. In patients who are symptomatic, bronchiectasis (22%) and chronic bronchitis (18%) are most commonly seen, with manifestations like necrotic nodules is seen in as few as 6% of the patients with symptomatic pulmonary EIM [11]. As patients can often be asymptomatic, pulmonary nodules can be found incidentally during evaluation for other medical ailments, posing as a red herring that may alter the clinician’s management of the original chief complaint. For this reason, understanding the complex and various EIM seen with IBD is imperative to the appropriate and timely treatment of patients with IBD.

We present the case of a 22-year-old African American female with a new diagnosis of UC whose course of care for appendicitis was complicated by the presence of numerous pulmonary nodules found during her evaluation.

## Case presentation

**Figure 1:**
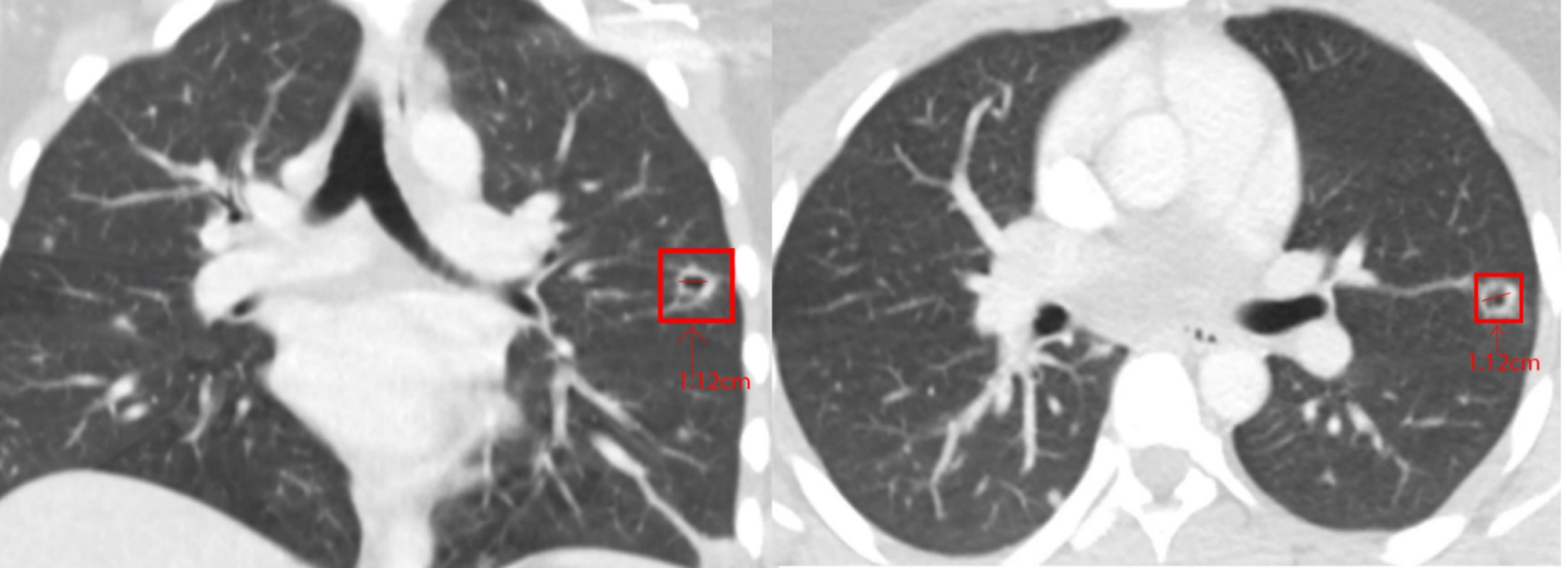
CT chest with contrast from outside hospital prior to admission showing multiple, bilateral cavitary nodules Left: coronal plane CT chest with contrast. Right: axial plane CT chest with contrast.

**Figure 2:**
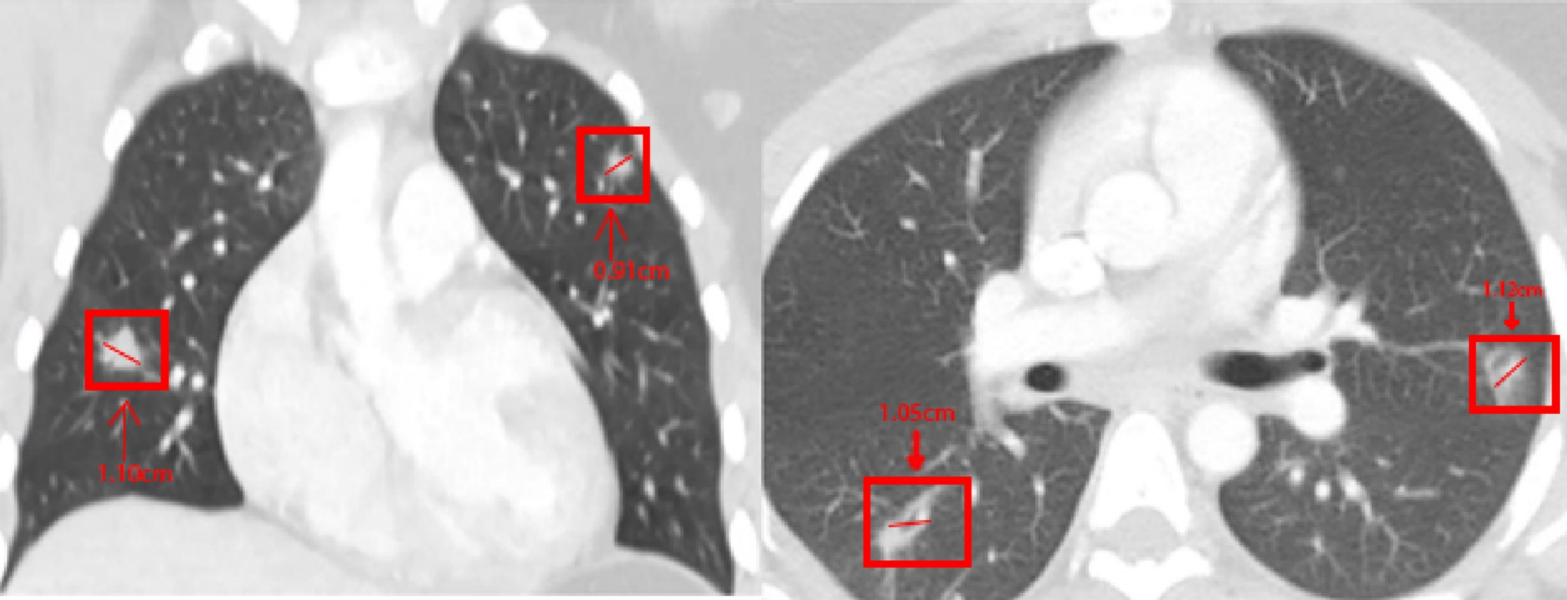
CT chest with contrast during admission with pulmonary nodules unchanged from prior CT imaging studies Left panel: axial CT chest. Right panel: coronal CT chest.

A 22-year-old African American female with a recent diagnosis of UC was transferred from an outside hospital (OSH) due to concerns for ruptured appendicitis. On preoperative imaging from the OSH, she was found to have bilateral pulmonary nodules and cavitating lesions. The ruptured appendicitis ultimately required surgical intervention after failing conservative therapy with intravenous piperacillin-tazobactam 3.375 g every six hours. She was evaluated by the pulmonary team regarding the abnormal lung imaging which revealed more than 10 pulmonary nodules in each lung, with the largest being ~2.1 cm in the right upper lobe (Figure 1). A repeat CT with contrast five days later confirmed multiple pulmonary nodules, with and without cavitation, present in all lobes (Figure 2). There was no mediastinal lymphadenopathy nor masses appreciated on either CT scan, with only residual thymic tissue noted in the anterior superior mediastinum.

The patient’s medical history included the recently diagnosed UC and a known left-sided ovarian teratoma. She states that at the time of her UC diagnosis she was experiencing multiple episodes of bloody diarrhea, polyarticular arthralgias in greater than five joints, and a sensation of “chest tightness” for six weeks. Her social history was negative for smoking, alcohol, or drug use. She denied recent travel or risk factors for other exposures. She has lived in New Jersey for her entire life, works in retail, with no chemical/animal exposures, or exposure to individuals with tuberculosis. Besides the chest tightness that she endorses, the patient denied hemoptysis, dyspnea, shortness of breath, orthopnea, cough, or sneezing. All other review of systems was negative.

Regarding her UC diagnosis, the patient had a colonoscopy with biopsies showing diffuse, continuous inflammation of the colonic mucosa. CT chest at the time of the colonoscopy and concurrent diagnosis (six weeks prior) noted the presence of multiple pulmonary nodules. She had not yet started treatment for the UC but stated that her bloody diarrhea had self resolved along with her polyarticular arthritis.

During the current admission, her vital signs were significant for fever and tachycardia. She was normotensive. She had no lymphadenopathy, her lungs were clear to auscultation bilaterally with normal inspiratory and expiratory effort, there were no wheezing, crackles, rales or rhonchi, and there was no dullness to percussion.

Laboratory testing was remarkable for an elevated prothrombin/international normalized ratio (17.3 and 1.6), partial thromboplastin time (42.0), elevated white blood cell count of 15.2 x 10^3^/µL (normal 4.5-11.00 x 10^3^ cells/µL), decreased hemoglobin of 8.9 g/dL (normal 12.0-14.0 g/dL), elevated alkaline phosphatase of 150 U/L (normal 39-117 U/L), and elevated lactate dehydrogenase 281 U/L (normal 110-230 U/L). Her beta-human chorionic gonadotropin (beta-HCG) was negative. Blood cultures, fungal cultures, hepatitis A/B/C panels, and HIV testing were all negative.

The pulmonary nodules were not believed to be septic emboli from the portal/mesenteric system despite a possible source of her known rupture appendicitis because the nodules were noted to predate the appendicitis by six weeks. Furthermore, the nodules were unchanged from four days of intravenous piperacillin-tazobactam at the OSH prior to her transfer. The nodules were not believed to be secondary to malignancy due to her negative beta-HCG, and only mild elevations in alkaline phosphatase and lactate dehydrogenase.

## Discussion

While EIM of UC are common, the severity of the EIM often correlates with disease activity [12]. Extraintestinal pulmonary involvement with necrotic nodules due to UC is incredibly uncommon, occurring in less than 6% of the patients with pulmonary involvement [11]. The presence of pulmonary nodules is even more rare when one considers the onset pulmonary EIM in patients with UC occurring after an average of 12 years from the time of initial IBD diagnosis [13]. The nodules are similar in their radiographic appearance to other, potentially life-threatening pathologies. In the case of our patient, the lack of extensive documentation and evaluation at the time of her initial diagnosis resulted in delay in surgical management of her appendicitis, multiple CT scans with contrasts of the chest, and alterations in the course of her care.

The differential diagnosis of pulmonary nodules is lengthy and complicated [14]. In a young patient, with a known infectious source (appendicitis) and ovarian mass (teratoma), pulmonary involvement of UC is appropriately low on the differential. Furthermore, when consideration is given to the age and race of the patient, sarcoidosis must be considered. Sarcoidosis is classically seen in young African American females, and can cause nodules similar to those seen in our patient [14]. Given the presence of her concurrent medical condition, the initial working diagnosis was septic embolization from the appendix, supported by the CT read indicating the findings to be suggestive for septic emboli, and evaluation for possible lung metastasis from a gynecological neoplasm was also initiated.

In consideration for metastatic disease, the size and appearance of the nodules offer radiological clues that must be analyzed carefully. The size of the nodules, ranging from 0.8 to 2.1 cm, can be classified as intermediate to high risk for malignancy (0.8-20 cm, and >20 cm). However, the remaining characteristics of the patient history and exam - a under 45-year old, nonsmoker, with no exposures to asbestos, prior history ofchronic obstructive pulmonary disease or other cancers - decrease the likelihood of malignancy [14]. In addition, the gynecological mass was present for several months, and outside records described similar measurements similar to those on ultrasound during her admission. Furthermore, in a patient who states she otherwise is feeling well, with no upper respiratory symptoms or risk factors, malignancy is unlikely, especially with other possible etiologies of the pulmonary nodules.

The initial working diagnosis was pulmonary nodules due to septic emboli, most likely based on the radiographic appearance of the nodules with a known source. Septic emboli are most commonly seen in patients with underlying valvular pathology and endocarditis but can occur as embolization from other sources within the body. Radiographically, the appearance of septic emboli is similar to the nodules present on the CT scans of our patient [10]. The nodules are present in multiple lobes of both lungs, wedge-shaped, with and without cavitation, and irregular. However, the clinical history of the patient did not support this diagnosis. The nodules were noted six weeks prior to her admission, there was no known source for embolization at that time, and the patient was well appearing, with no history of drug use or risk factors for septic embolization. Additionally, the pulmonary nodules demonstrated no change in response to appropriate antibiotic coverage. The workup for metastasis from gynecological neoplasm was unremarkable. Sarcoidosis was determined to be less likely due to the lack of hilar lymphadenopathy and other symptoms outside of pulmonary nodules suggestive of the diagnosis.

The presence of pulmonary nodules requires adjustments in the medical and surgical management of a patient, and further evaluation if the nodules are not previously well described. Depending on the etiology of the pulmonary nodules, patients may have impaired respiratory function, and be at an increased risk for operative complications due to anesthesia or intubation. Furthermore, the nodules may prompt as escalation in level of care requiring transfer to a higher-level facility and delaying definitive intervention for the chief complaint, as seen with our patient. The treatment of the nodules themselves is unique to the etiology; metastatic nodules, sarcoidosis, septic emboli, and extraintestinal manifestations of UC requiring vastly different treatments, which may harm a patient if the diagnosis is not correct. Treatment of pulmonary nodules secondary to malignancy can involve extensive surgical resection or immunologic/chemotherapeutic agents that can hinder the immune system. Pulmonary nodules resulting from UC require antimetabolite therapy and systemic corticosteroids [15]. Due to the difficulty in ruling out infectious etiology of the nodules, patients are often not treated with steroids, despite the benefit of steroids in patients with flares of their autoimmune disease [7,10,15].

The evaluation and management of pulmonary nodules in the setting of UC is based heavily on the clinical suspicion of the physician. Serial CT scans to track the nodules, assessing for interval change or regression, are commonly performed. Additional modalities such as biopsy of the nodules, pulmonary function testing, and bronchoscopy are also options for the evaluation of the nodules [15]. However, like other types of EIM, the pulmonary nodules tend to regress over time with treatment of the underlying IBD. Regardless of the evaluation and treatment strategy, the presence of the nodules must be well documented, and the patient educated on their disease in order to prevent complications or redundant and expensive testing in future episodes of care by new physicians.

## Conclusions

Patients with a history of IBDs, such as UC, may present with pulmonary nodules at the time of their diagnosis with UC. While pulmonary manifestations are rare, diligent evaluation and documentation of the etiology of the nodules is essential. Thorough evaluation of the nodules at time of presentation will facilitate appropriate diagnostic testing and treatment not only for the nodules themselves, but also for other unrelated illnesses the patient develops.
